# Did the speech of patients with Myasthenia Gravis decline over 4 years?

**DOI:** 10.1590/2317-1782/20232023055en

**Published:** 2023-11-10

**Authors:** Annelise Ayres, Marina Martins Pereira Padovani, Maira Rozenfeld Olchik, Maiara Laís Mallmann Kieling, Vanessa Brzoskowski dos Santos, Rui Rothe-Neves, Mara Behlau

**Affiliations:** 1 Centro de Estudos da Voz – CEV - São Paulo (SP), Brasil.; 2 Faculdade de Ciências Médicas da Santa Casa de São Paulo – FCMSCSP - São Paulo (SP), Brasil.; 3 Programa de Pós-graduação em Ciências Médicas, Universidade Federal do Rio Grande do Sul – UFRGS - Porto Alegre (RS), Brasil.; 4 Serviço de Neurologia, Hospital de Clínicas de Porto Alegre – HCPA - Porto Alegre (RS), Brasil.; 5 Curso de Fonoaudiologia, Universidade Federal do Rio Grande do Sul – UFRGS - Porto Alegre (RS), Brasil.; 6 Departamento de Cirurgia e Ortopedia, Faculdade de Odontologia, Universidade Federal do Rio Grande do Sul – UFRGS - Porto Alegre (RS), Brasil.; 7 Laboratório de Fonética da Faculdade de Letras, Universidade Federal de Minas Gerais – UFMG - Belo Horizonte (MG), Brasil.; 8 Departamento de Fonoaudiologia, Universidade Federal de São Paulo – USP - São Paulo (SP), Brasil.

**Keywords:** Speech, Voice, Dysarthria, Speech Acoustics, Myasthenia Gravis, Fala, Voz, Disartria, Acústica da Fala, Miastenia Grave

## Abstract

**Purpose:**

To compare the speech and voice patterns of myasthenia gravis (MG) patients over four years, and correlate the results with clinical aspects of the disease.

**Methods:**

Data was collected for 4 years. The clinical assessment tools included the Quantitative Myasthenia Gravis (QMG) score, the Myasthenia Gravis Foundation of America (MGFA) clinical classification, and the Myasthenia Gravis Quality of Life 15-item Scale (MG-QoL). To assess speech, the recorded speaking tasks were analyzed acoustically and given auditory-perceptual ratings. Sex (equal distribution) and age (p=0.949) were used as matching criteria in the final sample, which consisted of 10 individuals in the MG group (MGG) and 10 individuals in the control group (CG).

**Results:**

After 4 years, the MG participants presented stable health status, increased mild and moderate dysarthria (from 40% to 90% of the subjects), and a significant deterioration in the respiration, phonation, and articulation subsystems. The acoustic analysis showed a decline in articulatory patterns (speech rate p=0.047, articulation rate p=0.007, mean syllable duration p=0.007) and vocal quality (increased jitter p=0.022). In the follow-up comparison, there was a significant difference between the phonation variables (shimmer and harmonic-to-noise ratio) of the MGG and CG.

**Conclusion:**

The MG patients presented a decline in speech over four years and an increase in mild and moderate dysarthria. Despite presenting stable health status, their respiratory, phonatory, and articulatory subsystems worsened. There was no correlation between speech patterns and clinical characteristics of the disease (severity and motor scale).

## RESUMO

**Objetivo:**

Comparar o padrão de fala e voz de indivíduos com Miastenia Gravis (MG) em um intervalo de quatro anos e correlacionar com aspectos clínicos da doença.

**Método:**

A coleta de dados foi realizada ao longo de 4 anos. A avaliação clínica foi composta pelo Quantitative Myasthenia Gravis Score (QMGS), pela Myasthenia Gravis Foundation of America Classification (MGFA) e pela escala de qualidade de vida para Miastenia Gravis (MG-QOL). A avaliação da fala foi composta por gravação de tarefas, análise perceptivo-auditiva e análise acústica. A amostra final foi composta por 10 indivíduos em MG e 10 indivíduos no grupo controle (GC), pareados por sexo (distribuição igualitária) e idade (p=0,949).

**Resultados:**

Após 4 anos, os indivíduos com MG apresentaram estabilidade clínica, aumento do diagnóstico de disartria leve e moderada (de 40% para 90% dos sujeitos) e diminuição significativa no desempenho dos subsistemas da fala: respiração, fonação e articulação. Na análise acústica, houve declínio do padrão articulatório (taxa de fala p=0,047, taxa de articulação p=0,007, duração média das sílabas p=0,007) e qualidade vocal (jitter aumentado p=0,022). Houve diferença significativa nas variáveis fonatórias (*shimmer* e *harmonic-to-noise ratio*) entre os grupos MG e GC na comparação do seguimento.

**Conclusão:**

Indivíduos com MG apresentaram declínio no padrão de fala em um intervalo de quatro anos, com aumento no número de disártricos (leve e moderado). Mesmo com a estabilidade da doença, houve piora dos subsistemas respiratório, fonatório e articulatório. Não houve correlação entre o padrão de fala e as características clínicas da doença (gravidade e escala motora).

## INTRODUCTION

Myasthenia gravis (MG) is an autoimmune disease caused by pathogenic antibodies at the neuromuscular junction and impaired neuromuscular transmissions^(1-3)^. Although MG is an uncommon disease, the prevalence rate has increased over the past few years. Recent studies estimate a prevalence of 20 per 100,000 population in the US, 5.35 to 35 per 100,000 individuals worldwide, and an annual incidence ranging from 0.3 to 2.8 per 100,000 individuals^(4)^.

The main clinical manifestations of MG are weakness and fluctuating fatigue in the skeletal muscles which worsens with exercise and improves with rest. This weakness can cause varying degrees of ocular symptoms (diplopia and ptosis) and bulbar symptoms (dysarthria, dysphagia, difficulty chewing, weakness in the facial muscles, and poor breathing)^(2,3,5)^. Dysarthria is a common MG symptom. The prevalence of motor speech disorder as an early symptom ranges from 6 to 27%, and affects approximately 60% of patients as the disease progresses^(6-8)^. Dysarthria in MG is of the flaccid type, and is caused by fatigue and muscle weakness in speech organs such as the vocal folds, tongue, palate, and pharyngeal constrictors^(5,6,9)^.

The main complaints are hoarseness, vocal fatigue, poor pitch control and decreased loudness and projection^(5,6,9)^. Auditory-perceptual ratings have highlighted voice alterations such as hypernasality, poor pitch control, vocal fatigue, intermittent aphonia, stridor, a breathy or harsh vocal quality, irregular distribution of energy along the vocal tract, articulatory imprecision and breaks in verbal fluidity^(5,6,8-12)^.

The findings of acoustic analyses have shown a higher mean fundamental frequency, a higher mean fundamental frequency of the vibrating vocal folds, disturbance in jitter and shimmer values and the harmonic-to-noise ratio (HNR), higher mean duration of the silent interval between syllables during oral diadochokinetic tasks, and unstable spectrographic tracings characterized by absent harmonics at high frequencies^(5,6,8-12)^.

So far, few longitudinal studies have addressed MG symptoms and possible complications resulting from myasthenic exacerbation and crisis. One longitudinal study^(13)^ involving more than 1,000 English patients showed that, for most participants, serious disease-related events such as myasthenic exacerbation, myasthenic crisis or hospitalization had occurred in the first 2 to 3 years after diagnosis. This data suggests that treatment efficiency was achieved after this period. In addition, there was no difference between all-cause mortality scores in the MG group and controls during follow-up.

However, persistent symptoms like dysarthria are expected, even in patients with a stable health status. Yet the relationship between the pathophysiology of MG and speech disorders is still inconclusive. No longitudinal monitoring of speech in MG patients has produced evidence in the literature.

Therefore, the null hypothesis of this study was that there would be no change in the speech or voice patterns of MG patients over a 4-year period, given their stable status. The alternative hypothesis was that there would be changes over the 4-year period. The primary objective was to compare the speech and voice patterns of MG patients during the research period. The secondary objective was to correlate the voice and speech findings with clinical aspects of the disease (e.g., motor speech scales, age, education, and duration of illness), quality of life, and self-perception of speech changes.

## METHODS

### Study design

This was a longitudinal study. It was approved by the ethics committee of the Hospital de Clínicas de Porto Alegre (application number 120399), in compliance with the Declaration of Helsinki. All participants signed an informed consent form before assessments.

### Participants

Via telephone, MG patients who participated in a previous study^(8)^ were invited to participate in the new investigation. These individuals were follow-up outpatients at the tertiary referral clinic for neuromuscular diseases at the Hospital de Clínicas de Porto Alegre (HCPA), in Brazil. The study included native speakers of Brazilian Portuguese (age ≥ 18 years) with an MG diagnosis confirmed by electromyography and/or acetylcholine/MuSK/anti-striated muscle antibodies. Patients were excluded if they had a history of neurological events or smoking, sensory or motor disorders that could affect test performance, systemic diseases and/or structural alterations that affect the voice and/or speech, or benefitted from speech-language rehabilitation during the study period.

Sex and age were used as matching criteria to select a healthy control group (CG) with no correlation of familiarity. There was no test robust enough to assess the tasks under conditions of normality, so a control group was used to determine if variations detected during assessment were normal. All participants (MG and CG) spoke Brazilian Portuguese as their native language.

Baseline data were collected from February 2017 to December 2018. The second data collection phase occurred 4 years later (from September 2021 to December 2021). The initial sample consisted of 38 MG patients. There was a loss of 73.6% after the first assessment because 2.6% (1) died, 5.2% (2) moved to another city, 2.6% (1) declined enrollment, 26.3% (10) missed the evaluation and 36.8% (14) could not be reached via telephone. Sex and age were used as matching criteria for the final sample of 10 MG patients and 10 controls (CG).

There was no significant difference between the ages of MG Group (MGG) (follow-up) and CG (p=0.949). The mean time between the baseline assessment and follow-up was 50 months (±5 months). The baseline and follow-up data from MGG showed no significant difference in the motor speech or MG-related quality-of-life scores. Regarding speech self-perception, there was significant improvement (Table 1). A descriptive analysis of the clinical variables per MG subject, at baseline and follow-up, is presented in Chart 1.

**Table 1:** Sociodemographic data of the case and control groups

**Variables**	**MGG**		**CG**	**p**
	**Baseline**	**Follow-up**		
Age	52.50 (±17.29)	56.90 (±17.74)	56.40 (±16.60)	-
Duration of illness	10.90 (±4.87)	14.80 (±8.72)	-	-
Education	9.40 (±4.47)	10.0 (±4.87)	-	-
ROMP	14.30 (±6.48)	10.80 (±3.55)	-	0.017
MG-QOL	18.30 (±16.30)	17.70 (±18.38)	-	0.482
QMG	12.90 (±9.17)	13.20 (±6.98)	-	0.766
Male	5 (50)	5 (50)	5 (50)	-

Wilcoxon Signed Rank Test.

**Caption:** MGG = myasthenia gravis group; CG = control group; ROMP = Radboud Oral Motor Inventory for Parkinson's disease; MG-QoL = Myasthenia Gravis Quality of Life 15-Item Scale; QMG = The Quantitative Myasthenia Gravis score.

**Chart 1:** Description of the clinical variables of MG patients

**Subject**	**Sex**	**MGC**		**MGFA**		**Thymectomy**		**Immunoglobulin therapy**		**G**		**AI**		**IM**		**Hospital admissions due to myasthenic crisis**		**Antibody**	**COVID history**
		**1**	**2**	**1**	**2**	**1**	**2**	**1**	**2**	**1**	**2**	**1**	**2**	**1**	**2**	**1**	**2**		
001	M	0	7	2A	2A	-	-	-	-	+	-	+	+	-	+	-	-	**anti** -AChR +	-
002	F	19	19	1	2A	-	-	-	-	+	+	+	+	+	+	-	+	anti-MUSK - **anti** -AChR -	March/2021
003	F	25	26	2A	2A	-	-	-	-	-	-	+	+	-	-	-	-	no	February/2021
004	F	25	10	2B	2A	+	+	-	-	-	-	+	+	-	+	-	-	no	-
010	M	8	6	1	2A	-	-	-	-	+	-	-	+	+	+	+	-	**anti** -AChR +	-
012	F	2	13	2A	2A	+	+	-	-	-	-	+	+	+	-	-	-	no	-
016	F	13	19	2A	2A	+	+	-	-	-	-	+	+	-	-	-	-	**anti** -AChR +	-
019	M	14	9	2A	2A	+	+	+	-	-	+	+	+	+	+	-	-	**anti** -AChR +	-
023	M	4	5	1	1	-	-	-	-	-	-	+	+	+	+	-	-	no	-
031	M	9	19	2A	2A	-	-	-	-	+	-	+	+	-	-	-	-	**anti** -AChR +	July/2021

**Caption:** 1 = baseline; 2 = follow-up; M = male; F = female; + = underwent or uses; - = did not undergo or does not use; MGC = The Quantitative Myasthenia Gravis score; MGFA = The Myasthenia Gravis Foundation of America classification; G = glucocorticoids; AI = acetylcholinesterase inhibitors; IM = immune-modulating medications.

### Study procedure

The medical records of the MG patients were searched to collect clinical and sociodemographic data from their last hospital appointment before our study (e.g., age, sex, education, duration of illness, currently prescribed medication, surgical history, disease staging, motor symptoms, number of hospital admissions, COVID-19 history).

All participants were evaluated individually in a quiet, designated room at HCPA. The same trained administrator facilitated each visit. It took an average of 30 minutes for the MG subjects to complete the speech tasks and the following questionnaires:

- *Myasthenia Gravis Quality of Life 15-item Scale* (MG-QoL): a self-perception questionnaire specifically designed to assess the quality of life of MG patients. The score for each of the 15 items varies from 0 to 60 points. The higher the score, the worse the quality of life^(14,15)^.
- *Radboud Oral Motor Inventory for Parkinson's Disease* (ROMP): a self-perception questionnaire focused on speech, swallowing, and saliva complications. Only the 7-item domain of speech was used in our assessment. The score ranges from 7 to 28 points, and a lower score means fewer speech complaints^(16,17)^.
- *The Quantitative Myasthenia Gravis* (QMG) score: a clinical scale used as an MG outcome measure, with a maximum score of 39 points. It consists of 13 items. A higher score indicates more severe disease^(18,19)^.
  ● *The Myasthenia Gravis Foundation of America* (MGFA) classification: a clinical classification that groups patients into five progressively severe classes. Class I is characterized by “any ocular muscle weakness” with preserved strength in other muscles. Class V is defined by “intubation, with or without mechanical ventilation, except when used during routine postoperative management”^(20)^.

An Acer Aspire One 725-0899 computer, a KARSECT HT-9 headset microphone coupled to an Andrea PureAudio adapter and Audacity software were used to record and collect the speech samples from both groups. With the microphone positioned at 5 cm distance, the voice samples were recorded at 44.1 kHz with 16-bit resolution^(21)^. The following tasks were tested: maximum phonation time (MPT) – with the vowel /a/ sustained as long as possible after a deep inhalation, diadochokinetic (DDK) syllable rate - /pataka/, pitch variation – with the diphthong /iu/ produced several times in a single breath, automatic sequence (numbers 20 to 30), sentence imitation using 2 intonation patterns - “It rained a lot this weekend.” (statement), “Is she going on vacation?” (question) and “Today is my lucky day.”(exclamation), and spontaneous speech elicited by the question “ Which route did you take to get here?”. Patients were instructed to reply at their habitual pace and loudness.

### Auditory-perceptual and acoustic analysis

For the auditory-perceptual analysis of voice and speech, all audio files were edited and normalized using PRAAT software, version 6.1.11, and played for 3 blinded examiners. These speech-language therapists are members of the FONAD research group and have at least 5 years of experience evaluating and planning therapy for dysarthria. Prior training was carried out with audio files not used in the study. The Fleiss Kappa test was used to measure agreement, resulting in a score of k≥0.90 (excellent) for the variable of dysarthria. After listening to all the audio files once in random order, a consensus-based evaluation was performed. Upon request, audio files could be replayed. The speech subsystems (phonation, articulation, respiration, resonance and prosody) were analyzed based on the definitions described by Duffy^(9)^. The authors used a severity scale of 0 to 4 for motor speech changes (0 = normal, 1 = mild dysarthria, 2 = moderate dysarthria, or 3 = severe dysarthria).

Praat^(22)^ version 6.1.11 was used for the acoustic analysis. The following parameters of the prepared audio files were assessed, based on Rusz et al.^(23)^ and Vogel and Maruff^(24)^:

- Phonation - sustained vowel /a/: jitter (rap), shimmer (local), fundamental frequency (F_0_), standard deviation of F_0_, HNR.
- Articulation – DDK rate /pataka/ and spontaneous speech (MPT, speech rate, articulation rate, average syllable duration (ASD)).
- Respiration - sustained vowel /a/: MPT.
- Resonance - diphthong /iu/: the ratio between the 2nd vowel formant frequency of /i/ and the 2nd vowel formant frequency of /u/.
  ● Prosody – statement, exclamation, and question imitation: variations in frequency and intensity.

A specific automatic script^(25)^ was used for the articulation tasks to detect syllable nuclei in intensity peaks and automatically measure the diadochokinetic and spontaneous speech rates.

### Statistical Analysis

Descriptive data analysis was used to describe variable distributions. Absolute and relative frequencies were used to analyze categorical variables, and mean and standard deviation were used to analyze quantitative variables. The Wilcoxon signed-rank test was used to compare baseline and follow-up data from MGG. A bootstrap hypothesis test (Pearson's correlation coefficient) was used to analyze clinical variables and the follow-up results of MGG. Another bootstrap hypothesis test (Student's t-test) was used to compare the follow-up data from MGG and CG, and equalize independent sample means. The McNemar Test was used to compare the results of the auditory-perceptual assessment. Statistical significance was set at p<0.05. Results were statistically analyzed using version 18.0 of the Statistical Package for the Social Sciences (SPSS).

## RESULTS

A comparison between the baseline and follow-up MG data showed improvement in MPT after 4 years. This finding may be associated with the respiratory and prosody subsystems, since more significant variations in fundamental frequency were associated with statement sentences. However, there was a decline in articulatory performance. Patients produced fewer syllables per second, shorter syllable duration averages during spontaneous speech, and abnormal jitter thresholds (Table 2). There was a higher number of voices diagnosed with mild or moderate dysarthria due to altered phonation and articulation. This matched the articulatory and phonatory subsystem disruptions detected during follow-up (Table 3).

**Table 2:** Comparison between the acoustic variables of the baseline and follow-up MGG

**Variables**	**Baseline**		**Follow-up**		**Z**	**p**
	**Mean**	**SD**	**Average**	**SD**		
Phonation – sustained /a/ F_0_						
Mean	156.65	36.26	162.56	42.97	-1.070 b	0.285
SD	12.99	15.83	10.04	14.63	-.357 c	0.721
Minimum	137.20	45.62	134.77	49.52	-0.153 ^b^	0.878
Maximum	209.77	74.76	180.43	43.47	-0.459 ^c^	0.646
Shimmer (local)	9.28	4.49	12.77	9.53	-0.866 ^b^	0.386
Jitter (RAP)	0.33	0.33	0.64	0.75	-2.293 ^b^	0.022*
Articulation – diadochokinetic syllable rate						
Speech rate	4.41	1.52	4.65	1.55	-0.153 ^b^	0.878
Articulation rate	4.53	1.48	4.65	1.55	-0.051 ^b^	0.959
Mean syllable duration	0.25	0.13	0.25	0.14	-0.051 ^c^	0.959
Articulation - spontaneous speech						
Speech rate	3.71	0.36	3.26	0.4	-1.988 ^c^	0.047*
Articulation rate	4.84	0.53	3.85	0.47	-2.701 ^c^	0.007*
Mean syllable duration	0.20	0.02	0.26	0.03	-2.701 ^b^	0.007*
Respiration - sustained vowel						
MPT	5.22	2.72	11.84	6.30	-2.395 ^b^	0.017*
Resonance - diphthong						
F_2_ /i/	2219.75	578.57	2242.27	127.30	-1.682 ^b^	0.093
F_2_ /u/	802.92	149.12	1001.69	193.87	-1.784 ^c^	0.074
F_2_ /i/ / F_2_ /u/	2.81	0.85	2.30	0.40	-1.478 ^b^	0.139
Prosody - counting numbers						
Frequency variation	349.99	82.66	359.54	108.14	-1.376 ^b^	0.169
Prosody – statement						
Frequency variation	71.11	31.87	122.92	61.38	-2.947 ^b^	0.013*
Intensity Variation	29.35	8.16	27.36	5.72	-0.357 ^c^	0.721
Prosody – question						
Frequency variation	91.29	71.41	102.89	47.63	-0.764 ^b^	0.445
Intensity Variation	29.30	5.39	26.44	3.79	-0.968 ^c^	0.333
Prosody – exclamation						
Frequency variation	118.92	86.81	116.96	67.20	-0.561 ^b^	0.575
Intensity Variation	31.71	7.26	29.31	7.94	-0.663 ^c^	0.050

Wilcoxon Signed Rank Test.

* statistical significance set at p<0.05;

b negative ranks;

c positive ranks

**Caption:** MGG = myasthenia gravis group; SD = standard deviation; F_0_ = fundamental frequency; MPT = maximum phonation time.

**Table 3:** Auditory-perceptual analysis of MG patients

**Speech subsystem**	**Classification**	**Baseline**	**Follow-up**	**p**
		**N (%)**	**N (%)**	
Phonation	Normal	2 (20)	1 (10)	1.000
	Mild dysarthria	7 (70)	6 (60)	
	Moderate dysarthria	1 (10)	3 (30)	
Articulation	Normal	7 (70)	3 (30)	0.219
	Mild dysarthria	2 (20)	3 (30)	
	Moderate dysarthria	1 (10)	4 (40)	
Respiration	Normal	6 (60)	2 (20)	0.125
	Mild dysarthria	3 (30)	6 (60)	
	Moderate dysarthria	1 (10)	2 (20)	
Resonance	Normal	10 (100)	8 (80)	-
	Mild dysarthria	-	2 (20)	
Prosody	Normal	9 (90)	10 (100)	-
	Mild dysarthria	1 (10)	-	
Dysarthria severity	Normal	6 (60)	1 (10)	0.063
	Mild dysarthria	3 (30)	6 (60)	
	Moderate dysarthria	1 (10)	3 (30)	

McNemar Test.

**Caption:** MG = myasthenia gravis.

There was a statistical difference between the shimmer (local) and HNR thresholds of both follow-up groups. This suggests more significant irregularity in vocal fold vibration and a higher level of phonatory noise in the case group (Table 4).

**Table 4:** Comparison between the follow-up acoustic variables of MGG and CG

**Acoustic variables**	**MGG**	**CG**	**t**	**p**
	**Mean (SD)**	**Mean (SD)**		
Phonation - sustained vowel F_0_				
Mean	162.56 (±42.97)	167.51 (±48.98)	-0.240	0.813
Standard deviation	10.04 (±14.63)	10.98 (±19.57)	-0.122	0.904
Minimum	134.77 (±49.52)	137.17 (±56.91)	-0.101	0.921
Maximum	180.43 (±43.47)	183.84 (±58.36)	-0.148	0.884
Shimmer (local)	12.77 (±9.53)	5.05 (±2.58)	2.471	0.024*
Jitter (local)	1.09 (±1.23)	0.37 (±0.20)	1.809	0.087
HNR	12.62 (±8.32)	19.52 (±5.51)	-2.186	0.042*
Articulation - diadochokinetic syllable rate				
Phonation time	6.86 (2.81)	10.55 (5.23)	-1.962	0.070
Speech rate	4.65 (1.55)	5.02 (1.25)	-0.591	0.562
Articulation rate	4.65 (1.55)	5.18 (1.23)	-0.845	0.410
Mean syllable duration	0.25 (0.14)	0.20 (0.69)	0.950	0.360
Articulation - spontaneous speech				
Phonation time	28.27 (± 6.81)	31.50 (± 12.84)	-0.703	0.491
Speech rate	3.26 (± 0.41)	3.28 (± 0.57)	-0.072	0.944
Articulation rate	3.85 (± 0.47)	4.22 (± 0.67)	-1.411	0.177
Mean syllable duration	0.26 (± 0.03)	0.24 (± 0.03)	1.366	0.189
Respiration - sustained /a/				
MPT	11.84 (±6.30)	15.60 (±8.30)	-1.141	0.269
Resonance – Diphthong /iu/				
F_2_ /i/	2242.27 (±127.30)	2222.83 (±238.10)	0.228	0.822
F_2_ /u/	1001.69 (±193.87)	872.31 (±114.45)	1.817	0.086
F_2_ /i/ / F_2_ /u/	2.30 (±0.40)	2.5831 (±0.42)	-1.520	0.146
Prosody – Statement				
Frequency variation	122.92 (±61.38)	97.76 (±48.84)	1.014	0.325
Intensity variation	27.36 (±5.72)	31.51 (±3.39)	-1.974	0.064

Bootstrap hypothesis test (Student 's t-test)

* statistical significance set at p<0.05

**Caption:** MGG = myasthenia gravis group; CG = control group; SD = standard deviation; F_0_ = fundamental frequency; MSD = mean syllable duration; MPT = maximum phonation time; HNR = Harmonics-to-noise ratio.

No significant correlations were found between the acoustic findings and the clinical variables of the MGG (Table 5). The results suggest no relationship between clinical characteristics and a decline in speech patterns.

**Table 5:** Correlation between clinical variables and follow-up data from the MG group

	**Age**		**Duration of illness**		**ROMP**		**MGQoL**		**MGC**		**Shimmer (local)**		**HNR**	
	**r**	**p**	**r**	**p**	**r**	**p**	**r**	**p**	**r**	**p**	**r**	**p**	**r**	**p**
**Age**	-	-	0.403	0.248	-0.207	0.567	-0.309	0.384	0.146	0.687	0.553	0.098	-0.421	0.226
**Duration of illness**	0.403	0.248	-	-	-0.399	0.253	-0.476	0.164	-0.322	0.365	-0.027	0.940	0.017	0.963
**ROMP**	-0.207	0.567	-0.399	0.253	-	-	0.404	0.247	0.467	0.173	0.169	0.642	-0.093	0.799
**MG-QoL**	-0.309	0.384	-0.476	0.164	0.404	0.247	-	-	0.601	0.066	-0.388	0.268	0.528	0.117
**MGC**	0.146	0.687	-0.322	0.365	0.467	0.173	0.601	0.066	-	-	0.157	0.665	0.050	0.891
**Shimmer (local)**	0.553	0.098	-0.027	0.940	0.169	0.642	-0.388	0.268	0.157	0.665	-	-	-0.955	0.000*
**HNR**	-0.421	0.226	0.017	0.963	-0.093	0.799	0.528	0.117	0.050	0.891	-0.955	0.000*	-	-

Bootstrap Hypothesis Test (Pearson’s correlation coefficient)

* statistical significance set at p<0.05

**Caption:** r = Pearson's correlation coefficient; ROMP = Radboud Oral Motor Inventory for Parkinson's disease; MG-QoL =Myasthenia Gravis Quality of Life scale; MGS = The Quantitative Myasthenia Gravis score; HNR = Harmonics-to-noise ratio.

## DISCUSSION

This study investigated the speech and voice patterns of MG patients after a four-year interval and correlated the results with clinical aspects of the disease. This longitudinal study confirmed our alternative hypothesis. Although the MG patients presented a stable health status, there was a decline in speech performance and an increase in the number of participants diagnosed with mild or moderate dysarthria.

The auditory-perceptual analysis results showed worse respiration, phonation, and articulation. The acoustic analysis also detected a significant increase in jitter thresholds (phonation), a reduced number of syllables per second, and a shorter mean syllable duration during spontaneous speech (articulation). The respiratory results were more difficult to interpret since the auditory-perceptual analysis revealed an increase in participants with speech pattern alterations. Still, an objective measurement of MPT showed improvement between baseline and follow-up performance. Regarding prosody, in particular statement sentences, there was more frequency variation during follow-up.

There were no differences between the clinical scale and MG classifications. This was expected because of the pathophysiology of the disease^(1,3,13)^. However, the speech results were different. Phonatory and articulatory performances worsened, albeit mainly to a mild degree. Therefore, it is reasonable to hypothesize that speech intelligibility was not significantly affected, especially not with shorter utterances (sentences). There was no significant correlation between the acoustic analysis and the clinical variables in MGG.

The speech subsystem scores were lower than those of the first assessment^(8)^. Patients presented a further decline in the subsystems that were already altered at baseline. Phonation, respiration, and articulation were most affected, in the order of highest incidence. Resonance and prosody had not changed over the 4 years. Preserved resonance patterns are characteristic of the flaccid dysarthria associated with MG^(5,9,10)^. However, this aspect may change more in times of crisis and improve with clinical stabilization. Furthermore, Harris et al.^(13)^ demonstrated that MG symptoms could further deteriorate with drug use, such as the prolonged use of corticosteroids over time. This is why it is important to record MG patients’ vocal and articulatory patterns, regardless of the resonance quality.

In addition, the percentage of patients diagnosed with dysarthria increased from 40% to 90%. We found a higher prevalence than the literature (50 to 60% throughout the course of the disease)^(6-8)^.

Regarding the improvement in the MPT task and the intonation of statement sentences, our hypothesis is that there was inherent variation due to learning. Given that the clinical aspects of the disease remained stable and that the participants repeated the speech assessments, we believe familiarity may have influenced their performance. It should be noted that, despite improvement at follow-up, the MPT scores remained abnormal^(9,10,26)^. As for intonation, there is no current normative data for Brazilian Portuguese. Additionally, improved MPT and intonation of statement sentences did not assist phonatory performance in the MG patients.

When the follow-up MGG and CG were compared, there was a statistical difference regarding the control of voice intensity and the signal-to-noise ratio. The MG patients presented worse vocal quality. These were the parameters that distinguished the myasthenic patients from the controls. This finding is similar to results that other authors have described^(8-10,12)^. These are characteristics of the phonatory progression in MG.

The DDK rate task was not sensitive enough to detect articulatory decline in MG patients. This has been a controversial test in literature. Konstantopoulos et. al.^(12)^ described a higher mean duration of silent intervals between syllables as a dysarthric feature in MG. In a previous study of ours^(9)^, the DDK rate test was also not sensitive enough to differentiate the MG patients from the controls.

We have two hypotheses for this result. The first is: short tasks are easier for MG patients to execute without being affected by fatigue^(26)^. The second hypothesis is that the DDK rate task is better suited to assess speech motor programming^(27,28)^. Repeated syllable tests may be more sensitive to muscle fatigue and changes in speech patterns in MG patients MG.

There were no reported changes in the quality of life for the MG patients. Another longitudinal study^(29)^ with a significant number of patients in remission did not find any improvement in their quality of life after ten years.

Speech self-perception improved during the study period. The hypothesis is that, as the phonatory pattern slowly worsens over the years, MG patients adapt to these changes. Therefore, speech self-perception questionnaires should not be used as the only monitoring tool for dysarthria in this population, as it does not seem sensitive enough to identify changes over time.

Clinical services for MG patients lack appropriate referral networks for multidisciplinary follow-up. Frequently, MG patients are only referred to speech therapists after hospital admissions associated with a myasthenic crisis. Given that we detected speech disorders in patients with stable status, speech monitoring by therapists is important. MG patients require multidisciplinary care^30^. We recommend a minimum assessment protocol that tests a sustained vowel /a/, the DDK rate using the same syllable, and spontaneous speech (minimum time of 60 seconds) to evaluate vocal quality and articulatory patterns. The auditory-perceptual analysis of speech in our research corroborated the acoustic markers.

Losing 73.6% of the initial sample may have influenced the data and impacted other analyses, such as the correlations between speech symptoms and the use and dosage of medication. The high number of sample losses demonstrates the difficulty in recruiting patients for research in low-incoming countries where outpatient follow-up is complicated and difficult due to social, economic, and educational issues. Further longitudinal studies and larger samples of this population are necessary.

## CONCLUSION

The longitudinal analysis showed a decline in MG patients’ speech patterns, and a higher number of mild or moderate dysarthria diagnoses over four years. Despite having a stable health status, MG patients presented worse respiratory, phonatory, and articulatory performance. There was no correlation between speech patterns and the clinical characteristics of the disease (severity and motor scale), suggesting that the pathophysiology of the disease and speech in MG patients progress independently over time.
